# Genetic Diversity, Population Structure, and Marker-Trait Association for Drought Tolerance in US Rice Germplasm

**DOI:** 10.3390/plants8120530

**Published:** 2019-11-21

**Authors:** Uttam Bhattarai, Prasanta K. Subudhi

**Affiliations:** School of Plant, Environmental and Soil Sciences, Louisiana State University Agricultural Center, Baton Rouge, LA 70803, USA; uttambhattrai@gmail.com

**Keywords:** *Oryza sativa*, genetic diversity, marker trait association, moisture stress, population structure, reproductive stage

## Abstract

Drought is a major constraint in some rice-growing areas of the United States. Its impact is most severe at the reproductive stage resulting in low grain yield. Therefore, assessment of genetic and phenotypic variation for drought tolerance in US rice germplasm is necessary to accelerate the breeding effort. Evaluation of 205 US rice genotypes for drought tolerance at the reproductive stage revealed tolerant response in rice genotypes Bengal, Jupiter, Cypress, Jazzman, Caffey, and Trenasse. Harvest index and fresh shoot weight were identified as important traits to explain the majority of variability among the genotypes under drought tolerance. Genotyping with 80 SSR markers indicated a low level of genetic diversity in US germplasm. Population structure analysis grouped the genotypes into eight clusters. The genotypes from California, Louisiana, and Arkansas formed distinct subgroups. Texas genotypes were similar to those from Louisiana and Arkansas. Marker-trait association analysis showed significant association of RM570 and RM351 with grain yield, spikelet fertility, and harvest index whereas shoot dry weight showed association with RM302 and RM461. The drought-tolerant genotypes identified in this study and the SSR markers associated with drought tolerance attributes will be helpful for development of improved drought-tolerant rice varieties through marker assisted selection.

## 1. Introduction

Rice is a hydrophyte and requires a large amount of water for its growth and development. Since the majority of rice-producing areas in Asia and Africa are rainfed, moisture stress, particularly during reproductive stage, reduces yield drastically and threatens food security for millions of people. Drought in California and water restrictions in Texas have negatively impacted rice production in the United States. It shifted the rice acreage to other crops leading to a decline in rice production [1]. There is an urgent need to explore the available genetic resources for drought tolerance and understand the inherent tolerance mechanisms to expedite development of rice varieties for water stress environments.

Genetic diversity analysis helps to explore the variability present in rice germplasm for identification of desirable agronomic attributes. There are an estimated 140,000 diverse rice genotypes in the world. The IRRI germplasm center has preserved ~100,000 genotypes [2]. These genotypes have several desirable traits, which can be exploited for genetic enhancement. Identification of rice genotypes with high yield or yield stability under drought stress is an important prerequisite for breeding drought-tolerant varieties. Principal component analysis (PCA) and cluster analysis based on the phenotypic attributes can be used to assess the genetic variability [3]. Plant height, leaf rolling score, grain yield, spikelet fertility, and harvest index are some important traits commonly used for evaluating drought tolerance [4].

The environmental influence on phenotypic trait expression limits the utility of genetic diversity studies based on phenotypic traits. On the contrary, assessment of genetic variability using molecular markers is useful for crop improvement. Simple sequence repeat (SSR) markers have been extensively used to evaluate genetic diversity in rice [5,6]. The SSR markers are multi-allelic and can detect more genetic variation compared to single nucleotide polymorphism (SNP) [7] and amplified fragment length polymorphism (AFLP) markers [8]. Narrow genetic diversity in the US rice genotypes was observed in a previous study using SSR markers [9]. However, the study was based on cultivars released in the United States during the 20th century. A comprehensive study of the genetic diversity in all available US germplasm, including the recently developed genotypes, is necessary.

Population structure analysis is a model-based approach that classifies individuals into sub-populations. It helps to identify the admixture or migrants in a population [10]. Identifying the population structure before conducting association analysis reduces type I and type II errors, which may arise due to unequal allele frequency between sub-groups. Markers linked to the traits of interest can be identified using the principle of linkage disequilibrium i.e., non-random association between the alleles at different loci [6]. Compared with the bi-parental mapping approach, the association mapping accounts for the recombination events accumulated from the past several generations. Therefore, the results from such types of analyses reflect a larger amount of variation in the population.

There has been no comprehensive study on assessment of US rice genotypes for drought tolerance to date. The objectives of this study were: (i) to screen the US rice genotype collection for drought tolerance at the reproductive stage under greenhouse condition, (ii) to study the genetic variation and population structure in US rice germplasm, and (iii) to identify markers associated with yield and yield-contributing traits under drought stress.

## 2. Results

### 2.1. Assessment of Drought Tolerance Variability in US Rice Germplasm

Significant variations for all nine traits were observed among genotypes under drought stress (Table 1, Figure S1). The mean days to heading for all rice genotypes grown under the greenhouse condition was ~75 days. The average leaf rolling score after exposure to drought was quite high. The percentage of phenotypic variance explained by our data was greater than 60% for all the variables. There was a significant positive correlation between days to heading and yield-related traits (Table 2), which could be due to production of new flowering panicles in previously matured tillers during the drought recovery period contributing toward yield. The number of tillers was negatively correlated with grain yield and harvest index because the new tillers produced from the plants during recovery period did not mature to contribute towards yield. Significant and moderate correlations between leaf rolling score and yield-related traits were observed. Shoot fresh weight did not correlate significantly with yield. However, shoot dry matter content was significantly and negatively correlated with grain yield and other yield-related traits.

**Figure 1:**
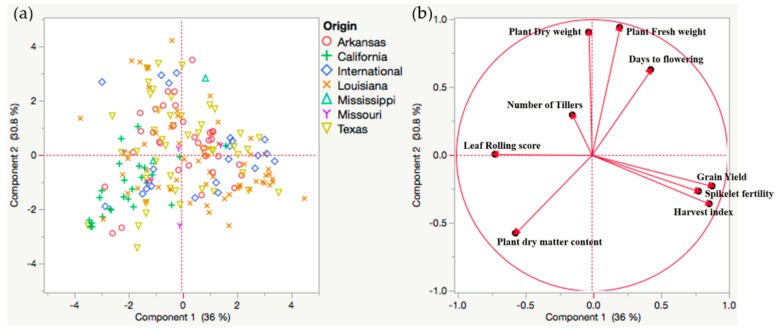
Principal component analysis (PCA) plot of various agronomic traits, yield, and yield-related traits in the US rice genotypes. (**a**) Scatter plot of the various rice genotypes represented in two major principal component axes. No sufficient clustering was observed except the California genotypes in the third quadrant. (**b**) Grouping of the variables in two principal components. PC1 represented yield-related traits and PC2 represented the agronomic traits.

Principal component analysis was done to identify the grouping pattern among the genotypes based on agronomic and yield-related traits (Figure 1a). The first two principal components accounted for 36% and 31% of the genetic variance (Figure 1b). Three principal components with eigen values greater than 1 explained 83% of variability in the data set (Table S1). Allocation of variables to its principal components is a subjective approach and was based on its principal component score. A cut off value of 0.35 indicated four variables contributed to principal components 1 and 2 and one variable contributed to principal component 3 (Table S2). Principal components were not correlated among each other indicating different principal components were responsible to explain the variability in a different set of variables. Principal component 1 (PC1) accounted for the contrast among the rice genotypes for grain yield, spikelet fertility, harvest index with their positive co-efficient and leaf rolling score with their negative coefficient. Similarly, PC2 represented the variation for shoot-related traits *viz.* shoot fresh weight, shoot dry weight with a positive coefficient and shoot dry matter content with a negative coefficient. Principal component 3 explained variation among the rice genotypes for number of tillers. The most representative variables were harvest index and shoot fresh weight, which represented 73% and 68% of the variability in each group of traits, respectively. PCA did not show any distinct clustering of the US genotypes based on the origin of the state. This could be due to the fact that the principal component analysis was based on the level of drought tolerance (PC1 and PC2) rather than the variability among the genotypes due to their geographical origin. However, most of the genotypes from California were clustered in quadrant 3.

Based on nine phenotypic traits under drought stress (Table 3), genotypes were classified into six clusters (Table 4, Figure S2). The level of tolerance of each cluster was assessed by mean values of grain yield and yield-related traits such as spikelet fertility and harvest index. Therefore, the six clusters did not follow the order based on the level of drought tolerance. The genotypes clustered in group 6 were highly tolerant to drought stress. The mean grain yield, spikelet fertility, and harvest index of genotypes in group 6 were 10.3 g/plant, 54.4%, and 0.28, respectively. Group 6 included drought-tolerant check genotypes (Dular, SLO16, and Kalia), salt-tolerant genotype ‘Geumgangbyeo’, and medium grain US genotype ‘Bengal’. It also included many genotypes from Louisiana, Texas, Arkansas, and California. The moderately tolerant genotypes were in group 3. It included drought-tolerant checks (N-22, Chengri, Djogolon, and Pin Kaeo) and some US rice genotypes (Newbonnet, Cypress, and Caffey). Mean spikelet fertility, grain yield, and harvest index for the genotypes in this group were 46%, 6.3g/plant, and 0.22, respectively. Cluster 4 included tolerant rice genotypes: Early prolific, Rexona, Hybrid mix, and Jefferson. The spikelet fertility, grain yield, and harvest index of the genotypes in this group were 25%, 4 g/plant and 0.11, respectively. Clusters 1, 2, and 5 contained the genotypes that were susceptible to drought with mean grain yield of 2.5, 3, and 1.5 g/plant, respectively. Cluster 5 contained popular genotypes, Alan, Terso, and Tauri, which were most susceptible to drought.

### 2.2. Genetic Variability in US Rice Germplasm

Among the 80 SSR markers used for genotyping, five markers (RM7187, RM192, RM126, RM116, and RM512) were monomorphic (Table S3). The maximum number of alleles was 6 for RM8085. A total of 272 alleles were observed with an average of 3.4 alleles per marker. The major allele frequency for the polymorphic markers ranged between 0.39–0.97, with an average of 0.74. The genetic diversity of the markers varied from 0.05 (RM598) to 0.66 (RM8219). Polymorphism information content (PIC) among the polymorphic markers ranged between 0.05 (RM598) to 0.58 (RM488, RM8219, and RM 3428).

**Figure 2:**
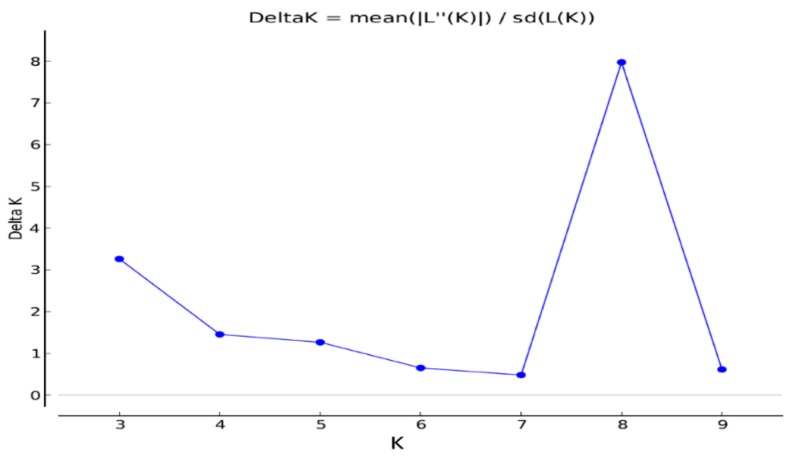
Estimation of population structure using LnP(D) derived ΔK for determining the optimum number of subpopulations. The maximum value of delta K was found to be at K = 8, suggesting division of the entire population into eight subpopulations.

**Figure 3:**
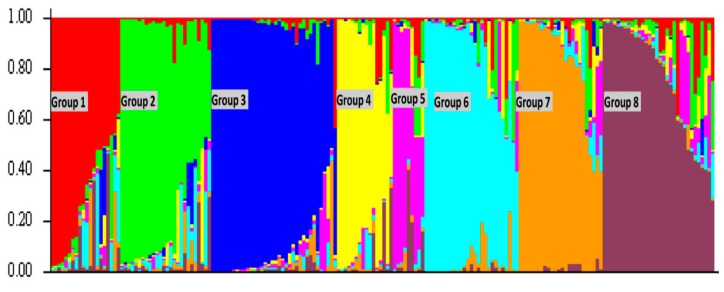
Assignment of US rice germplasms into eight populations using STRUCTURE 2.2 software. The y-axis corresponded to the subgroup membership and the x-axis represented the genotype. The genotypes with the probability of ≥70% were assigned to a specific subgroup, while the others were classified as admixtures.

The population structure of the rice genotypes was analyzed with the software ‘STRUCTURE’ using Bayesian clustering method. The membership fractions of 2–10 were used to classify the genotypes. The log likelihood LnP (D) and Evanno’s deltaK identified eight distinct clusters of the population (Figure 2 and Figure 3). The subgroup 1 (SG1) contained 12 genotypes belonging to *japonica* subspecies. The other genotypes clustered in this sub-group were admixtures. It contained genotypes from Arkansas and Texas. SG2 contained 19 genotypes and some admixtures. This subgroup was mostly dominated by the rice genotypes from Texas. All genotypes in SG2 were of *japonica* subspecies. SG3 contained the check genotypes and salt-tolerant lines obtained from the International Rice Research Institute (IRRI). They were mostly of *indica* subspecies. A few Louisiana genotypes and two weedy rice genotypes obtained from Mississippi (MS-1995-15 and MS-1996-9) were clustered in this subgroup. Fourteen genotypes were in SG4. All of them, except Moroberekan and R-27, were from Louisiana. SG5 contained four genotypes (Delitus-1206, Evangeline, Nira, and Leah) from Louisiana. SG6 contained 26 genotypes from Texas and Louisiana. All sixteen genotypes in SG7 were from Louisiana except Pin Kaeo and Kalia. Among the 20 genotypes in SG8, three (Arkose, Asahi, and Kamrose) were from Arkansas and the others were developed in California.

In the Unweighted pair group method with arithmetic mean (UPGMA) clustering, Louisiana genotypes were separated from other US rice genotypes (Figure S3). The check genotypes obtained from IRRI were highly diverse and did not cluster together with the US genotypes. This validated the findings from population structure analysis which suggested difference between US rice germplasm and the imported *indica* rice germplasm. Besides this, there were two major clusters that separated US rice germplasm. One cluster contained mostly the Louisiana rice germplasm and the second cluster contained rice germplasm from the remaining rice growing states of USA. The rice genotypes from Arkansas, California, and Texas were included in second cluster. However, California genotypes were separated from other US genotypes within the subgroup.

Although population structure analysis and UPGMA clustering differentiated genotypes into different groups, algorithms used in both software classified the genotypes based on the geographical origin. The three groups of US rice genotypes in UPGMA method included the groups of the rice genotypes from (i) Louisiana, (ii) California and (iii) Others (Texas and Arkansas). A similar grouping of genotypes was found in population structure analysis. Three broad groups divided by UPGMA clustering were sub-divided in population structure analysis based on their relatedness. The group of rice genotypes from Louisiana was divided into subgroup (SG) 4, SG5, and SG7. Similarly, the group of rice genotypes from California was placed in SG8 in population structure analysis. The rice genotypes clustered in Arkansas and Texas were separated into SG1, SG2, and SG6.

Eight subgroups obtained by ‘STRUCTURE’ were analyzed for the significant genetic differentiation between and among the groups. Analysis of molecular variance (AMOVA) revealed 42% and 58% of the total variation among the groups, and within the group, respectively (Table 5). The variation within the group and the variation among the groups were significantly different.

### 2.3. Marker Trait Associations

Generalized linear model (GLM) and mixed linear model (MLM) revealed 43 and 24 marker-trait associations for nine yield and agronomic traits, respectively (Table 6). Three marker trait associations were detected for days to heading in GLM method and there were two associations in MLM method. RM22 and RM3471 were associated with days to heading in both methods. RM168, associated with number of tillers, contributed only 3% of the phenotypic variance (PV). GLM and MLM together detected four markers (RM129, RM351, RM256, and RM216) linked to leaf rolling score. RM351 in chromosome 7 showed a strong association with 6% of PV for the leaf rolling score. Besides these, GLM detected RM129, RM152, and RM216, which contributed 5% of PV for the same trait. Five markers were significantly associated with shoot fresh weight in both GLM and MLM methods. RM302 in chromosome 1 contributed 5% of PV for shoot fresh weight. For dry weight, GLM and MLM detected 10 and 4 markers, respectively. RM302, RM3471, RM461, and RM8207 explained 9%, 6%, 8%, and 7% of the PV, respectively for shoot dry weight in GLM. RM315 showed association with shoot dry matter content in both methods. Both GLM and MLM detected four markers associated with spikelet fertility whereas GLM detected five and two markers associated with grain yield and harvest index, respectively. RM 570 located on chromosome 3 was associated with both spikelet fertility in GLM and harvest index in both methods. RM351 showed a strong association with harvest index with a phenotypic variance of 6% and 7% by GLM and MLM methods, respectively.

## 3. Discussion

Exploring the genetic and phenotypic variability in available germplasm resources is the first step in any successful breeding program. Drought is becoming a major threat to crop production, especially in rice, due to disturbances in world climate. However, there has been little effort to investigate drought tolerance in the US rice germplasm. Drought stress increases the days to flowering and reduces the plant height and spikelet fertility resulting in yield loss [4,11,12,13]. Leaf rolling is an important indicator for measuring drought responsiveness in rice [14,15]. A high average leaf rolling score of US rice germplasm indicated their susceptibility under drought. Correlation studies indicated that leaf rolling score negatively affected grain yield and spikelet fertility in rice. Therefore, early selection for yield could be done by scoring the leaf rolling under drought stress. Low spikelet fertility observed under drought stress was responsible for the drastic reduction in grain yield and harvest index. The yield reduction of >50% under drought stress was reported in earlier studies [16,17,18]. A wide variation in yield, spikelet fertility, and harvest index suggested that the US rice germplasm collection includes both drought-tolerant and susceptible genotypes.

There was a large variability for drought tolerance among the US rice germplasm (Figure 1). Drought tolerance among rice genotypes was not correlated with the state of origin, except for the rice genotypes from California. Most of the California genotypes were separated from the rice genotypes from other states and were more susceptible to drought. Principal component analysis further clarified that the three representative variables from each of the principal components (harvest index, shoot fresh weight, and number of tillers) were sufficient to capture most of the variation in the data. This could be due to low correlation values among these three variables and high correlation values among the variables within each of the principal components. These three traits could be used to screen the rice genotypes for drought tolerance. The variables in the first two principal components were more indicative of drought stress as the variables within those principal components were extremely affected by terminal drought stress. Furthermore, the US rice genotypes were grouped according to their level of drought tolerance. Tolerant genotypes had higher spikelet fertility and grain yield under drought stress compared to those of the susceptible ones. Traditional drought-tolerant genotypes like Dular, N-22, and Kalia [19] were grouped under tolerant category. The rice genotypes from various states grouped under the drought-tolerant category included Zenith, Mars, Arkrose, Asahi, Katy, Taggert, Magnolia, Wells, Templeton (from Arkansas), Dawn, Madison, Hill Long Grain (from Texas), MO R-500 (from Missouri), Rey, Della, Acadia, Saturn, Bengal, Dellmati (from Louisiana). The inclusion of CL142, CL111, and Mermentau under the drought-tolerant group in our study agreed with an earlier study [20]. Grouping of two known deep rooting genotypes, Moroberekan and Azucena, as drought susceptible could be due to low depth of soil in our pot experiment. Few salt-tolerant rice genotypes like Damodar, Cypress, Caffey, Jupiter, and Jazzman [21] showed drought tolerance under the greenhouse conditions. The rice genotypes showing both salt and drought tolerance may be due to similar physiological responses and co-expression of the genes under both stress conditions [22].

Genetic relatedness among the rice genotypes from six major rice growing states of the US was assessed using SSR markers, which were proven to be effective in identifying small allelic variation [7]. The average polymorphism information content (PIC) of 0.33 observed in our study was less compared to the average PIC of 0.54 in the global rice collection [23]. The PIC value with a range of 0.21–0.50 was observed in the US rice in previous studies [8,9]. A lower PIC value indicated the presence of low genetic diversity in US rice germplasm [3,8]. The genetic diversity of *japonica* subspecies was small compared to its *indica* counterpart. The average PIC of the global *japonica* rice collection was 0.42 and the European collection of tropical *japonica* rice was 0.37 [24]. The dominance of *japonica* subspecies in US rice germplasm collection may be the reason for low genetic diversity compared to the global collection.

Population structure analysis identified eight subgroups in the US germplasm (Figure 3). Two to eight subpopulations were reported in earlier studies [3,6,25,26]. The threshold to assign a genotype into a specific subgroup varied from 60 to 80% similarity. Our stringent threshold of 70% similarity identified 49 genotypes as admixtures. The check genotypes of *indica* group were separated from the *japonica* US genotypes. The rice genotypes from California, Louisiana, and Arkansas were different from each other, whereas the rice genotypes from Texas appeared to be a mixture of the genotypes from Louisiana and Arkansas. Few rice genotypes from Arkansas matched closely with the California rice genotypes. These findings led to the classification of the US rice genotypes into three major groups depending on the state of origin i.e., Louisiana, Arkansas, and California. The differences in rice genotypes from these three major rice-growing states may be due to different rice growing ecosystems prevailing in those regions. A similar study in US weedy rice showed variation in the rice genotypes according to the region of origin [27]. The result of the model-based structure analysis agreed with the results from the UPGMA tree (Figure S3). It indicated that the US rice genotypes were completely different from the Asian rice genotypes [9]. The two weedy rice genotypes from Mississippi (MS-1995-15 and MS1996-9) were closer to the *indica* genotypes.

Marker-trait association studies conducted in germplasm collection have been useful to identify the molecular markers linked to traits of interest [17,26]. Several markers, which were earlier reported to be colocalized with genes or QTLs, were linked to important agronomic traits in this study. For example, RM22, associated with days to heading under drought stress, was located on the same region of chromosome 3 as previously identified QTLs *qDTF3.01* [13], *DTY3.2* [28] and *hd9* [29]. RM216 co-localized with the previously identified QTL *qDTY10.1* [16]. The co-localization of RM302 associated with shoot fresh weight and shoot dry weight with the previously identified QTL for leaf water content suggested dependence of plant biomass on leaf water content [30]. RM246 linked to heading date was associated with the photosynthetic rate in rice suggesting relationship between both traits [30]. The leaf rolling score linked marker RM152 was associated with leaf water content and stomatal conductance in rice under drought stress [30]. Both plant biomass and leaf water content were highly correlated with drought tolerance in rice. The two novel markers, RM8207 and RM461, identified in this study could be useful for improving shoot dry weight under stress. Vikram et al. [28] earlier concluded that colocation of flowering and grain yield QTLs under drought was due to tight linkage. Although markers for this trait can be used to make indirect selection for grain yield, focus should also be made to use markers associated to spikelet fertility, grain yield and harvest index. RM431, linked to *qDTY1.1* [31], was associated with spikelet fertility and RM351 on chromosome 7 was associated with spikelet fertility, grain yield, and harvest index. Both RM523 and RM570 were linked to grain yield and harvest index in rice. These novel markers associated with grain yield and yield components could be useful for marker-assisted breeding to improve drought tolerance in rice.

## 4. Methods

### 4.1. Plant Materials and Drought Tolerance Screening

The present study material consisted of 205 rice accessions of which 138 were obtained from the National Genetic Resource Program (NRGS), 38 from the Louisiana Rice Research Station (LRRS), and 29 from the International Rice Research Institute (IRRI). The accessions obtained from IRRI originated from 14 different countries around the world. The remaining 176 accessions were cultivars developed by U.S. rice breeding programs with 56 cultivars originating from Louisiana, 47 from Texas, 38 from Arkansas, 26 from California, seven from Missouri, and two from Mississippi. The details of the accessions used in the experiment and their states of origin were listed in Table S4.

The rice genotypes were screened for drought tolerance at the reproductive stage under greenhouse condition. The experiment was conducted in a complete randomized design (CRD) with two replications. Plants were grown in 7.5-liter plastic pots filled with silty clay soil. Three plants were allowed to grow in each pot under ambient conditions with no moisture stress until the emergence of the panicles. A total of six plants per genotype were sampled for analysis. Since there was wide variation in days to panicle initiation among the genotypes, plants of each genotype were monitored every day. Once a plant showed panicle initiation, it was removed from the watered bench and placed in a concrete bench without water for one week. The temperature inside the greenhouse during the plant growth period was maintained 35 ℃/29 ℃ day/night regime and the relative humidity of 70%–80% was maintained by the mist generating pad at the wall of the greenhouse chamber. During the panicle initiation stages of the genotypes, 13.5 to 14 h of daylight was observed. One week of drought stress under the greenhouse conditions during the panicle initiation stage induced >50% spikelet sterility in majority of the genotypes. Some susceptible genotypes experienced irreversible damage as evidenced from extremely low grain yield. After exposing the plants to drought stress for one week without irrigation, the plants were moved to a bench with water. Observations were taken on various morphological and yield-attributing traits to assess the level of drought tolerance in rice. Days to heading (DTH) was measured as the number of days from planting to the emergence of the first panicle in the main tiller. Number of tillers (NT) in each plant was counted. Leaf rolling score (LRS) was given following the protocol from standard evaluation system of rice in the scale of 1–9 [32]. The leaves below the flag leaf were visually examined during morning hours to avoid artificial wilting due to high temperature to get the leaf rolling score. LRS was evaluated at 5 and 7 days after drought stress and averaged to get the composite score for each genotype. Shoot fresh weight (SFW) was measured as the fresh weight of the above-ground plant. Shoot dry weight (SDW) was measured after drying the plant samples in oven at 65 ℃ for one week. Shoot dry matter content (SDMC) was measured as the ratio of shoot dry weight to shoot fresh weight and expressed in percentage. Spikelet fertility (SF) was calculated as the ratio of the number of fertile spikelets to the total number of spikelets in a plant and expressed in percentage. Grain yield (GY) was measured in each of the three plants and averaged. Harvest index (HI) was calculated as the ratio of grain yield to shoot dry weight.

### 4.2. Genotyping

One hundred eighty-four genotypes were genotyped using 80 SSR markers. DNA was isolated from young leaf tissues of each genotype using Cetyl trimethylammonium bromide (CTAB) method [33]. Quantification of DNA was done using spectrophotometer (Nanodrop ND-1000, Thermofisher Scientific, Waltham, MA, USA). A final DNA concentration of 50 ng/µL was used for PCR amplification. The PCR reaction mixture contained 3 µL of 50 ng/µL DNA, 12.8 µL of water, 2.5 µL of 10x PCR mixture, 2.5 µL each of 25 mM MgCl_2_ and 2 mM dNTPs, 1.25 µL 50 ng/µL of both forward and reverse primers, and 1 U of Taq polymerase (Promega Corporation, Madison, WI, USA). The PCR reaction profile consisted of 35 cycles of the following steps: denaturation at 94 ℃ for 45 s; annealing at 55 ℃ for 45 s (varied depending on the SSR marker); and extension at 72 ℃ for 1 min with a final extension at 72 ℃ for 5 min. The annealing temperature used for PCR reactions was obtained from the Gramene database (http://archive.gramene.org/markers/). The PCR products were run in 4.5% super fine resolution (SFR) agarose gel and were viewed under UV using gel documentation system.

### 4.3. Statistical Analysis

The mean values of the two replications were used for analysis. Mean range and r-square value for various agronomic and yield traits were calculated in SAS using proc univariate. Correlations among the traits were calculated using Proc Corr procedure in SAS [34]. Principal component analysis (PCA) was used to study the relationship among the genotypes and to identify the important variables contributing to the phenotype. Cluster analysis was used to group the genotypes based on the phenotypic traits. PCA and cluster analysis was done using JMP [35]. The level of drought tolerance on various clusters was determined based on yield-related traits (spikelet fertility, grain yield per plant, and harvest index).

### 4.4. Genetic Diversity and Population Structure Analysis

The presence and absence of the alleles were scored as 1 and 0, respectively. The expected band size of the PCR products was obtained from the Gramene website (http://www.gramene.org/marker/). Gene diversity, average number of alleles per locus (AL), major allele frequency (MAF), and polymorphism information content (PIC) were calculated using ‘PowerMarker’ software V 3.5 [36]. Average number of alleles is the mean of the alleles present in all genotypes for a specific marker. Major allele frequency is the relative frequency of the most common allele for a particular marker. PIC was calculated as follows:

$$PIC_{i}=1-\overset{n}{\underset{i=1}{\sum }}P_{i}^{2}$$

where, ‘*i*’ is the *i*th allele of the *j*th marker, n is the number of alleles at the *j*th marker and *P* is the allele frequency

‘STRUCTURE 2.2′ was used to assess the population structure [10]. The parameters were set to 50,000 burns-in period followed by 50,000 Markov Chain Monte Carlo simulations. It allowed the admixtures and correlated allele frequencies. The genotypes were classified into sub-populations based on its maximum membership probability. A genotype was considered to be in a sub-population if >70% of its composition came from that group, otherwise, it was classified as an admixture. The optimum number of sub-population (K) was determined by running K values from 2 to 10. Each K value was run ten times. True value of K was determined using *adhoc* statistics ΔK proposed by Evanno et al. [37] in ‘Structure harvester’ [38].

The unweighted pair group method with arithmetic mean (UPGMA) clustering was done using ‘DARwin’ software [39]. Dissimilarity matrix used for constructing the tree was computed using a shared allele index. The Analysis of molecular variance (AMOVA) among the sub-populations identified by structure was computed using GenAlex V 6.5 with 1000 permutations [40].

### 4.5. Marker-Trait Associations

Association of markers with traits was determined using generalized linear model (GLM) and mixed linear model (MLM) in TASSEL 5 [41]. A significant marker trait association was declared when the *p*-value was less than 0.05.

## 5. Conclusions

Drought is a major limitation to rice production in United States and other parts of the world. Although rice is predominantly grown in lowland, well-watered conditions, disturbance in the global climate is limiting the availability of water for its successful cultivation. Since drought tolerance studies in the US rice germplasm are limited, the drought-tolerant US genotypes identified in this study will be useful for breeding drought-tolerant rice varieties. Identified drought-tolerant USA rice genotypes can be hybridized with the locally adapted genotypes to increase the genetic diversity of drought-tolerant rice cultivars in USA and other parts of the world. A low genetic diversity observed in US rice germplasm calls for the introduction of the new diverse germplasm to enhance genetic diversity. The molecular markers that were associated with the grain yield and yield contributing traits under drought stress will facilitate marker-assisted breeding to develop varieties with enhanced yield and stability in drought-prone areas.

## Figures and Tables

**Table 1:** Mean, range and r-square value for various agronomic traits, yield, and yield-related traits in rice genotypes under drought stress.

Traits	Mean	Range	R-square ^a^
Days to heading	74.9	62–88	0.94
Number of tillers	4.0	2.5–7	0.69
Leaf rolling score ^b^	6.2	3–9	0.73
Shoot fresh weight (g/plant)	86.4	29.2–230.4	0.88
Shoot dry weight (g/plant)	38.8	25–93.5	0.91
Shoot dry matter content (%)	47.9	13.9–90.0	0.76
Spikelet fertility (%)	30.4	0.1–90.0	0.88
Grain yield (g/plant)	5.7	0.1–46.3	0.87
Harvest index	0.15	0.001–0.62	0.88

^a^ Amount of variation explained by the genotypes for a specific trait; ^b^ Leaf rolling score was measured in the scale of 1–9, 1 being highly tolerant and 9 is highly susceptible.

**Table 2:** Pearson correlation coefficients among various agronomic traits, yield, and yield-related traits in rice genotypes under drought stress.

	DTH	NT	LRS	SFW	SDW	SDMC	SF	GY	HI
DTH	1.00	−0.06	−0.19 **	0.55 **	0.53 **	−0.39 **	0.19 **	0.23 **	0.14 *
NT		1.00	−0.07	0.29 **	0.25 **	−0.25 **	−0.11	−0.13 *	−0.17 **
LRS			1.00	−0.17 **	0.07	0.52 **	−0.37 **	−0.45 **	−0.46 **
SFW				1.00	0.84 **	−0.66 **	−0.07	0.03	−0.11
SDW					1.00	−0.30 **	−0.21 **	−0.13 *	−0.26 **
SDMC						1.00	−0.21 **	−0.28 **	−0.22 **
SF							1.00	0.68 **	0.70 **
GY								1.00	0.93 **
HI									1.00

** Significant at 0.001 level of probability; * Significant at 0.01 level of probability; DTH, Days to heading; NT, Number of tillers; LRS, Leaf rolling score; SFW, Shoot fresh weight (g/plant); SDW, Shoot dry weight (g/plant); SDMC, Shoot dry matter content (%); SF, Spikelet fertility (%); GY, Grain yield (g/plant); HI, Harvest index.

**Table 3:** Mean value of each group identified by cluster analysis for agronomic traits, yield, and yield-related traits in the US rice genotypes under drought stress.

Group ^a^	Count ^b^	DTH	NT	LRS	SFW	SDW	SDMC	SF	GY	HI
1 (S)	36	83.0	3.8	6.8	121.4	52.8	44.7	15.3	2.5	0.06
2 (MS)	37	70.3	4.0	6.9	77.3	33.7	47.5	15.1	3.0	0.08
3 (T)	22	70.1	4.0	6.8	57.2	32.0	57.7	46.0	6.3	0.22
4 (MT)	17	72.4	6.0	6.0	97.0	42.5	47.1	25.4	4.0	0.11
5 (HS)	17	66.5	3.3	8.0	39.4	28.7	73.7	13.1	1.5	0.05
6 (HT)	68	77.2	3.7	4.9	91.6	37.9	42.2	54.4	10.3	0.28

^a^ Six different groups identified by cluster analysis: susceptible (S), moderately susceptible (MS), tolerant (T), moderately tolerant (MT), highly susceptible (HS), and highly tolerant (HT); ^b^ Number of rice genotypes in each group; DTH, Days to heading; NT, Number of tillers; LRS, Leaf rolling score; SFW, Shoot fresh weight (g/plant); SDW, Shoot dry weight (g/plant); SDMC, Shoot dry matter content (%); SF, Spikelet fertility (%); GY, Grain yield (g/plant); HI, Harvest index.

**Table 4:** Classification of the US rice genotypes for drought tolerance based on various agronomic traits, yield, and yield-related traits under drought stress.

Group (Level of Tolerance)	List of Rice Genotypes
Group 1 (Susceptible)	Starbonnet-1, Rexark-1, Starbonnet-2, Bluebonnet, Toro, Nova, Glutinous selection, FL378, Melrose, Arkansas fortune, Prelude, Rexark Rogue-9262, RD, Nova-66, Stormproof, Carolina Gold, Rexark-2, Lady wright, Sierra, Zenith-2, Epagri, C-4, Tokalon, Texas Patna, TP-49, Moroberekan, Lacrosse, Salvo, Delitus-120, Delitus, Rexark Rogue-9214, Nira-43, Nira, Cheriviruppu, Pokkali
Group 2 (Moderately Susceptible)	Bond, CL162, Tebonnet, S-201, Calrose, Gulfrose, Early Colusa, Vista, M-202, Cheniere, M-102, Jackson, Azucena, LA-0702086, R-52, Sabine, M-301, Calrose-2, Conway, M-201, Catahoula, Bluebelle, Vegold, Bluebelle-2, Pacos, Caloro, MS-1995-15, Gold Zenith, Brazos, Smooth Zenith, Newrex, Kamrose, Colusa, Family-24, Nato, Calady, Skybonnet
Group 3 (Tolerant)	Newbonnet, Cypress, Jazzman-2, Jodon, R-50, Pin Kaeo, N-22, Trenasse, Presidio, Kokubelle, Lafitte, Mermentau, Dixieblle, Palmyra, Rico-1, Early Wataribur, Maybelle, Della-2, Chengri, Kalia-2, Djogolon, Caffey
Group 4 (Moderately Tolerant)	Early Prolific, MS-1996-9, CL261, Hybrid Mix, Lebonnet, Lotus, Damodar, Rexona, S-301, CL111, M-204, CL131, R27, Neches, Lavaoa, Bellemont, Jefferson
Group 5 (Highly Susceptible)	Alan, Terso, Tauri Mai, M-103, Carlpearl, Maxwell, Nipponbare, M-401, Belle Patna, Earlirose, M302, Cocodrie, Millie, Texmont, Gody, Rossmont, Adair
Group 6 (Highly Tolerant)	Zenith, Mars, Arkose selection, Saturn Rouge, Della, Hill Long Grain, Nortai, Cody, Jasmine-85, Evangeline, Dawn, Asahi, Rey, Acadia, CR5272, Saturn, SLO16, Northrose, Bengal, Dellamti, Katy, Taggert, FL478, Lacarus, CL152, MO R-500, Arkose, Gold Nato, Earl, LAH10, LA0802140, CL181, Wells, Templeton, TCCP-266, CL161, Glutinous Zenith, Hill medium, Magnolia, R54, Century Rogue, Toro-2, Short Century, Century Patna, SP14, Orion, CSR-11, Jupiter, Mercury, Dellrose, Geumgangbyeo, CL142, Madison, R-609, Roy J, Neptune, Lacassine, Pirogue, Dellmont, Jazzman, Leah, IRRI147, Ecrevisse, PSVRC, Dular, Jes, Kalia, LA110

**Table 5:** Analysis of molecular variance (AMOVA) among the eight sub-populations identified by ‘STRUCTURE’ software.

Source of Variation	DF ^a^	SS ^b^	MSS ^c^	Estimated Variance	% variance	*P*-value^c^
Among Population	7	790.77	112.96	6.34	42	<0.0001
Within Population	124	1095.75	8.84	8.84	58	<0.0001
Total	131	1886.52		15.18	100	

^a^ Degree of freedom; ^b^ Sum of squares; ^c^ Mean sum of squares; ^d^ Level of significance.

**Table 6:** Significant marker trait association in rice genotypes under drought stress using GLM (Q) and MLM (Q+K) model.

Traits	Marker	Chr.	Pos. (Mb)	GLM ^a^ (Q) Model			MLM ^b^ (Q+K) Model		
				F-value	*P*-value ^c^	R-square ^d^	F-value	*P*-value ^c^	R-square ^d^
Days to heading	RM246	1	27.3	6.46	0.01	0.03			
	RM22	3	1.5	8.12	<0.01	0.03	7.61	0.01	0.04
	RM3471	4	6.3	10.25	<0.01	0.05	4.87	0.03	0.03
No. of Tillers	RM168	3	28.1	4.80	0.03	0.03	4.16	0.04	0.03
Leaf rolling score	RM129	1	19.0	9.23	<0.01	0.05	4.26	0.04	0.02
	RM168	3	28.1	7.69	0.01	0.04			
	RM570	3	35.6	8.35	<0.01	0.04			
	RM351	7	23.9	10.53	<0.01	0.06	5.25	0.02	0.04
	RM152	8	0.7	10.60	<0.01	0.05			
	RM256	8	24.3	4.54	<0.01	0.02	5.51	0.02	0.03
	RM216	10	5.4	11.27	<0.01	0.05	4.38	0.04	0.02
	RM7195	12	9.9	4.68	0.03	0.03			
Shoot fresh weight	RM302	1	33	10.27	<0.01	0.05	10.1	<0.01	0.05
	RM431	1	38.9	6.08	0.01	0.03	6.81	0.01	0.04
	RM3471	4	6.3	5.50	0.02	0.03	4.32	0.04	0.02
	RM289	5	7.8	4.72	0.03	0.02	5.62	0.02	0.03
	RM5371	6	25.8	6.06	0.01	0.03	4.22	0.04	0.02
	RM1376	8	3.2	5.25	0.02	0.03			
Shoot dry weight	RM129	1	19.0	5.98	0.02	0.03			
	RM302	1	33.0	20.95	<0.01	0.09	5.74	0.02	0.04
	RM14980	3	13.9	7.17	0.01	0.03			
	RM570	3	35.6	7.58	0.01	0.03			
	RM3471	4	6.3	11.72	<0.01	0.06	8.43	<0.01	0.05
	RM289	5	7.8	6.39	0.01	0.03	6.82	0.01	0.04
	RM5371	6	25.8	7.54	0.01	0.03			
	RM461	6	30.1	9.77	<0.01	0.08	8.45	<0.01	0.09
	RM351	7	23.9	8.37	<0.01	0.04			
	RM8207	10	9.8	7.48	0.01	0.07			
Shoot dry matter content	RM315	1	36.7	6.60	0.01	0.03	6.27	0.01	0.04
Spikelet fertility	RM431	1	38.9	6.09	0.01	0.03	4.44	0.04	0.03
	RM168	3	28.1	5.23	0.02	0.03			
	RM570	3	35.6	11.76	<0.01	0.06	7.25	0.01	0.04
	RM6054	5	22.8	8.95	<0.01	0.04	7.17	0.01	0.04
	RM351	7	23.9	6.68	0.01	0.04	5.02	0.03	0.03
	RM216	10	5.4	5.29	0.02	0.03			
Grain yield	RM523	3	1.3	5.38	0.02	0.03			
	RM517	3	6.2	5.50	0.02	0.03			
	RM570	3	35.6	5.80	0.02	0.03	4.12	0.04	0.03
	RM351	7	23.9	6.56	0.01	0.04			
	RM256	8	24.3	5.47	0.02	0.03			
Harvest Index	RM523	3	1.3	5.41	0.02	0.03			
	RM570	3	35.6	5.27	0.02	0.03	4.04	0.04	0.03
	RM351	7	23.9	10.51	<0.01	0.06	5.23	0.02	0.07

^a^ Generalized linear model; ^b^ Mixed linear model (MLM accounts for the population structure and kinship matrix); ^c^ Level of significance; ^d^ Variance contributed by the marker.

## Supplementary Materials

The following are available online at https://www.mdpi.com/2223-7747/8/12/530/s1, Figure S1: Frequency distribution of the various agronomic traits, yield, and yield-related traits in rice genotypes under drought stress, Figure S2: Clustering of rice genotypes based on nice phenotypic traits under drought stress, Figure S3: Unweighted pair group method with arithmetic mean (UPGMA) tree of rice genotypes using SSR markers. Different color codes represented rice genotypes developed in various states in the United States. Check genotypes were developed in different countries of the world and were obtained from IRRI, Table S1: Eigen vectors and eigen values of the principal components for various agronomic traits, yield, and yield-related traits in rice genotypes under drought stress, Table S2: Representative groups of traits identified by PCA analysis of various agronomic traits, yield, and yield-related traits in rice genotypes under drought stress, Table S3: Details of SSR markers used in genotyping of the rice genotypes, major allele frequency, number of alleles, genetic diversity, and PIC values, Table S4: List of the rice genotypes used in the experiment, their source of origin and subspecies category. The sub-group was determined by software ‘Structure’.
