# Supplementary material for: Genetic Diversity, Population Structure, and Marker-Trait Association for Drought Tolerance in US Rice Germplasm
Source: Plants (Basel). 2019 Nov 21;8(12):530. doi: 10.3390/plants8120530 (PMC6963191; doi:10.3390/plants8120530)
Supplement: Supplementary file 1 [file plants-08-00530-s001.zip › New Supplementary tables.docx]

**Table S1** Eigen vectors and eigen values of the principal components for various agronomic traits, yield, and yield-related traits in rice genotypes under drought stress

| Variable | PC1 | PC2 | PC3 | PC4 | PC5 | PC6 | PC7 | PC8 | PC9 |
| --- | --- | --- | --- | --- | --- | --- | --- | --- | --- |
| Eigen value | 3.24 | 2.77 | 1.08 | 0.76 | 0.40 | 0.35 | 0.28 | 0.05 | 0.04 |
| Variation (%) | 36.05 | 30.81 | 12.04 | 8.49 | 4.46 | 3.97 | 3.17 | 0.54 | 0.47 |
| Cumulative | 36.05 | 66.86 | 78.90 | 87.39 | 91.85 | 95.82 | 98.98 | 99.53 | 100.00 |
| DTF | 0.24 | 0.38 | 0.42 | 0.04 | 0.62 | 0.25 | -0.42 | 0.03 | 0.05 |
| NT | -0.08 | 0.18 | -0.80 | 0.44 | 0.25 | 0.18 | -0.16 | <0.01 | 0.02 |
| LRS | -0.40 | 0.01 | 0.29 | 0.59 | -0.17 | -0.47 | -0.41 | -0.01 | 0.04 |
| SFW | 0.11 | 0.57 | <0.01 | 0.04 | -0.27 | -0.10 | 0.22 | 0.52 | 0.52 |
| SDW | -0.01 | 0.54 | 0.16 | 0.29 | -0.18 | 0.17 | 0.41 | -0.48 | -0.36 |
| SDMC | -0.31 | -0.35 | 0.27 | 0.40 | 0.18 | 0.51 | 0.40 | 0.24 | 0.20 |
| SF | 0.44 | -0.16 | <0.01 | 0.28 | 0.44 | -0.56 | 0.44 | <0.01 | -0.01 |
| GY | 0.49 | -0.14 | 0.05 | 0.30 | -0.34 | 0.19 | -0.16 | 0.46 | -0.51 |
| HI | 0.48 | -0.22 | 0.02 | 0.23 | -0.29 | 0.19 | -0.16 | -0.48 | 0.54 |

PC, Principal components; DTF, Days to flowering; NT, Number of tillers; LRS, Leaf rolling score; SFW, Shoot fresh weight (g/plant); SDW, Shoot dry weight (g/plant); SDMC, Shoot dry matter content (%); SF, Spikelet fertility (%); GY, Grain Yield (g/plant); HI, Harvest index (%)

**Table S2** Representative groups of traits identified by PCA analysis of various agronomic traits, yield, and yield-related traits in rice genotypes under drought stress

| Groups | No. of variables  in each group | Most representative variable | Variation within cluster (%) | Overall variation (%) |
| --- | --- | --- | --- | --- |
| 1 | 4 | Harvest index | 0.73 | 0.32 |
| 2 | 4 | Shoot fresh weight | 0.68 | 0.30 |
| 3 | 1 | No. of tillers | 1.00 | 0.11 |

**Table S3** Details of SSR markers used in genotyping of the rice genotypes, major allele frequency, number of alleles, genetic diversity, and PIC values

| Marker | Chr. | Position^a^ (Mb) | Repeat motif^b^ | Product size^c^ (bp) | Major allele frequency | No. of alleles | Genetic diversity | PIC^d^ |
| --- | --- | --- | --- | --- | --- | --- | --- | --- |
| RM259 | 1 | 7.4 | (CT)17 | 162 | 0.81 | 3 | 0.33 | 0.29 |
| RM493 | 1 | 12.2 | (CTT)9 | 211 | 0.73 | 3 | 0.43 | 0.38 |
| RM466 | 1 | 17.2 | (AG)17 | 230 | 0.59 | 3 | 0.56 | 0.49 |
| RM129 | 1 | 19 | (CGG)8 | 205 | 0.83 | 3 | 0.30 | 0.27 |
| RM9 | 1 | 23.3 | (GA)15GT(GA)2 | 136 | 0.78 | 4 | 0.36 | 0.33 |
| RM488 | 1 | 24.8 | (GA)17 | 177 | 0.53 | 5 | 0.63 | 0.58 |
| RM246 | 1 | 27.3 | (CT)20 | 116 | 0.82 | 6 | 0.33 | 0.31 |
| RM302 | 1 | 32.9 | (GT)30(AT)8 | 156 | 0.81 | 4 | 0.33 | 0.30 |
| RM212 | 1 | 33 | (CT)24 | 136 | 0.87 | 4 | 0.24 | 0.23 |
| RM8085 | 1 | 34.8 | (AG)26 | 126 | 0.53 | 6 | 0.58 | 0.49 |
| RM315 | 1 | 36.7 | (AT)4(GT)10 | 133 | 0.76 | 3 | 0.39 | 0.35 |
| RM431 | 1 | 38.8 | (AG)16 | 251 | 0.80 | 4 | 0.34 | 0.31 |
| RM104 | 1 | 40.1 | (GA)9 | 222 | 0.66 | 4 | 0.48 | 0.41 |
| RM84 | 1 | NA | (TCT)10 | 113 | 0.87 | 4 | 0.23 | 0.22 |
| RM110 | 2 | 1.3 | (GA)15 | 156 | 0.80 | 4 | 0.33 | 0.30 |
| RM174 | 2 | 7 | (AGG)7(GA)10 | 208 | 0.58 | 3 | 0.56 | 0.48 |
| RM550 | 2 | 12.4 | (CCT)8 | 231 | 0.69 | 3 | 0.47 | 0.42 |
| RM262 | 2 | 20.7 | (CT)16 | 154 | 0.86 | 3 | 0.25 | 0.23 |
| RM13600 | 2 | 24.2 | (AG)11 | 122 | 0.61 | 5 | 0.57 | 0.51 |
| RM263 | 2 | 25.8 | (CT)34 | 199 | 0.72 | 4 | 0.45 | 0.40 |
| RM240 | 2 | 31.4 | (CT)21 | 132 | 0.73 | 4 | 0.44 | 0.40 |
| RM211 | 2 | NA | (TC)3A(TC)18 | 161 | 0.85 | 4 | 0.26 | 0.25 |
| RM327 | 2 | NA | (CAT)11(CTT)5 | 213 | 0.78 | 4 | 0.36 | 0.34 |
| RM60 | 3 | 0.1 | (AATT)5AATCT(AATT) | 165 | 0.75 | 4 | 0.40 | 0.36 |
| RM7332 | 3 | 0.4 | (ACAT)11 | 205 | 0.69 | 2 | 0.43 | 0.34 |
| RM523 | 3 | 1.3 | (TC)14 | 148 | 0.59 | 4 | 0.58 | 0.53 |
| RM22 | 3 | 1.5 | (GA)22 | 194 | 0.65 | 3 | 0.47 | 0.38 |
| RM569 | 3 | 1.9 | (CT)16 | 175 | 0.54 | 4 | 0.61 | 0.54 |
| RM517 | 3 | 6.1 | (CT)15 | 266 | 0.67 | 3 | 0.45 | 0.36 |
| RM14980 | 3 | 13.9 | (AG)17 | 382 | 0.84 | 3 | 0.27 | 0.24 |
| RM16 | 3 | 23.1 | (TCG)5(GA)16 | 181 | 0.71 | 4 | 0.45 | 0.41 |
| RM168 | 3 | 28.0 | T15(GT)14 | 116 | 0.8 | 5 | 0.34 | 0.32 |
| RM570 | 3 | 35.5 | (AG)15 | 208 | 0.45 | 4 | 0.64 | 0.57 |
| RM335 | 4 | 0.7 | (CTT)25 | 104 | 0.57 | 4 | 0.61 | 0.56 |
| RM3471 | 4 | 6.3 | (CT)21 | 147 | 0.59 | 3 | 0.57 | 0.51 |
| RM6314 | 4 | 18.4 | (CTT)11 | 169 | 0.82 | 3 | 0.30 | 0.26 |
| RM6250 | 4 | 24.8 | (CTC)8 | 187 | 0.95 | 2 | 0.10 | 0.09 |
| RM7187 | 4 | 27.4 | (ATAG)7 | 157 | 1.00 | 1 | 0.00 | 0.00 |
| RM437 | 5 | 3.8 | (AG)13 | 275 | 0.86 | 3 | 0.24 | 0.23 |
| RM289 | 5 | 7.8 | G11(GA)16 | 108 | 0.86 | 3 | 0.25 | 0.23 |
| RM598 | 5 | 16.7 | (GCA)9 | 159 | 0.97 | 3 | 0.05 | 0.05 |
| RM6054 | 5 | 22.7 | (CCG)12 | 128 | 0.78 | 4 | 0.34 | 0.30 |
| RM274 | 5 | 26.8 | (GA)15-7-(CGG)5 | 160 | 0.84 | 3 | 0.28 | 0.26 |
| RM587 | 6 | 2.3 | (CTT)18 | 217 | 0.77 | 3 | 0.38 | 0.34 |
| RM3 | 6 | 19.4 | (GA)2GG(GA)25 | 145 | 0.87 | 6 | 0.23 | 0.23 |
| RM5371 | 6 | 25.8 | (TC)13 | 143 | 0.76 | 3 | 0.38 | 0.34 |
| RM461 | 6 | 30.1 | (AAAC)6 | 195 | 0.52 | 3 | 0.56 | 0.46 |
| RM314 | 6 | NA | (GT)8(CG)3(GT)5 | 118 | 0.95 | 3 | 0.10 | 0.10 |
| RM192 | 7 | 0.2 | (TGG)5 | 267 | 1.00 | 1 | 0.00 | 0.00 |
| RM3449 | 7 | 13.4 | (CT)19 | 179 | 0.72 | 3 | 0.44 | 0.4 |
| RM5793 | 7 | 17.4 | (AGC)8 | 127 | 0.85 | 4 | 0.27 | 0.26 |
| RM351 | 7 | 23.9 | (CCG)9(CGAAG)4 | 134 | 0.71 | 3 | 0.45 | 0.40 |
| RM172 | 7 | 29.5 | (AGG)6 | 159 | 0.51 | 3 | 0.56 | 0.46 |
| RM152 | 8 | 0.6 | (GGC)10 | 151 | 0.72 | 4 | 0.44 | 0.4 |
| RM1376 | 8 | 3.1 | (AG)31 | 199 | 0.59 | 4 | 0.58 | 0.54 |
| RM515 | 8 | 20.2 | (GA)11 | 211 | 0.52 | 3 | 0.61 | 0.53 |
| RM256 | 8 | 24.2 | (CT)21 | 127 | 0.93 | 3 | 0.13 | 0.12 |
| RM126 | 8 | NA | (GA)7 | 171 | 1.00 | 1 | 0.00 | 0.00 |
| RM8219 | 9 | 1.5 | (GA)11 | 169 | 0.39 | 3 | 0.66 | 0.58 |
| RM6475 | 9 | 12.8 | (GCC)9 | 209 | 0.67 | 4 | 0.51 | 0.47 |
| RM566 | 9 | 14.7 | (AG)15 | 239 | 0.6 | 5 | 0.58 | 0.53 |
| RM107 | 9 | 20 | (GA)7 | 189 | 0.84 | 3 | 0.28 | 0.26 |
| RM6707 | 9 | 22.2 | (TAT)8 | 113 | 0.79 | 3 | 0.33 | 0.28 |
| RM6862 | 9 | NA | (TGC)9 | 113 | 0.78 | 2 | 0.34 | 0.28 |
| RM216 | 10 | 5.3 | (CT)18 | 146 | 0.66 | 5 | 0.51 | 0.46 |
| RM8207 | 10 | 9.8 | (TTC)23 | 191 | 0.52 | 4 | 0.63 | 0.58 |
| RM596 | 10 | 15.2 | (GAC)10 | 188 | 0.67 | 2 | 0.44 | 0.35 |
| RM258 | 10 | 18 | (GA)21(GGA)3 | 148 | 0.85 | 3 | 0.27 | 0.24 |
| RM3451 | 10 | 21.5 | (CT)19 | 208 | 0.77 | 4 | 0.37 | 0.33 |
| RM271 | 10 | NA | (GA)15 | 101 | 0.82 | 4 | 0.31 | 0.29 |
| RM26045 | 11 | 1.8 | (TC)12 | 297 | 0.49 | 3 | 0.59 | 0.51 |
| RM116 | 11 | 5.7 | (CT)9 | 258 | 1.00 | 1 | 0.00 | 0.00 |
| RM3428 | 11 | 13.4 | (CT)18 | 156 | 0.42 | 3 | 0.65 | 0.58 |
| RM209 | 11 | 17.8 | (CT)18 | 134 | 0.6 | 4 | 0.58 | 0.53 |
| RM7277 | 11 | 24.2 | (ATCT)10 | 148 | 0.83 | 2 | 0.29 | 0.25 |
| RM7187 | 11 | NA | (AT)29(GT)7 | 146 | 0.94 | 4 | 0.11 | 0.11 |
| RM20 | 12 | 0.9 | (ATT)14 | 140 | 0.86 | 3 | 0.25 | 0.23 |
| RM512 | 12 | 5.1 | (TTTA)5 | 214 | 1.00 | 1 | 0.00 | 0.00 |
| RM7195 | 12 | 9.9 | (ATAG)7 | 138 | 0.73 | 4 | 0.44 | 0.40 |
| RM5609 | 12 | 23.9 | (AAG)9 | 158 | 0.84 | 3 | 0.28 | 0.26 |
| Mean |  |  |  |  | 0.74 | 3.4 | 0.37 | 0.33 |

^a^Physical position of the marker in the chromosome in megabase (Mb); ^b^Repeat sequence of the SSR marker; ^c^Expected size of the PCR product in base pair (bp); ^d^Polymorphism information content

**Table S4** List of rice genotypes used in the experiment, their source of origin, subtype and the sub-group classification by software ‘STRUCTURE’

| S.N. | Genotype | Source^a^ | Sub-type^b^ | Sub-group^c^ | S.N. | Genotype | Source | Sub-type | Sub-group |
| --- | --- | --- | --- | --- | --- | --- | --- | --- | --- |
| 1 | Hasawi | Saudi Arabia | *Indica* | NG | 36 | Jazzman | Louisiana | *Japonica* | AD |
| 2 | Cheriviruppu | India | *Indica* | SG3 | 37 | Neptune | Louisiana | *Japonica* | SG4 |
| 3 | Pokkali | Srilanka | *Indica* | SG3 | 38 | Caffey | Louisiana | *Japonica* | SG4 |
| 4 | Nona Bokra | India | *Japonica* | NG | 39 | Templeton | Arkansas | *Japonica* | SG6 |
| 5 | Capsule | Bangladesh | *Indica* | NG | 40 | Taggert | Arkansas | *Japonica* | SG6 |
| 6 | FL478 | Philippines | *Japonica* | SG3 | 41 | Jazzman-2 | Louisiana | *Japonica* | AD |
| 7 | FL378 | Philippines | *Japonica* | SG3 | 42 | Jes | Arkansas | *Indica* | SG3 |
| 8 | TCCP-266 | Philippines | *Indica* | SG3 | 43 | CL162 | Louisiana | *Japonica* | SG3 |
| 9 | IRRI147 | Philippines | *Indica* | SG3 | 44 | CL181 | Louisiana | *Japonica* | SG7 |
| 10 | Epagri | Brazil | *Indica* | SG3 | 45 | CL111 | Louisiana | *Japonica* | SG7 |
| 11 | Damodar | India | *Indica* | AD | 46 | CL131 | Louisiana | *Japonica* | SG7 |
| 12 | Chengri | Bangladesh | *Indica* | SG3 | 47 | Cypress | Louisiana | *Japonica* | SG7 |
| 13 | CSR11 | India | *Indica* | SG3 | 48 | CL161 | Louisiana | *Japonica* | SG7 |
| 14 | PSVRC | - | *Indica* | SG3 | 49 | LA0702085 | Louisiana | *Japonica* | SG7 |
| 15 | Pin Kaeo | Thailand | *Indica* | SG7 | 50 | CL261 | Louisiana | *Japonica* | SG4 |
| 16 | Dular | Bangladesh | *Indica* | SG3 | 51 | N-22 | India | *Indica* | SG3 |
| 17 | Moroberekan | Guinea | *Japonica* | SG4 | 52 | CR5272 | Costa Rica | *Indica* | SG3 |
| 18 | Nipponbare | Japan | *Japonica* | AD | 53 | Agami | Egypt | *Indica* | NG |
| 19 | Geumgangbyeo | South Korea | *Indica* | SG3 | 54 | Arang | Indonesia | *Indica* | NG |
| 20 | IR-29 | Philippines | *Indica* | NG | 55 | Kalia-2 | Bangladesh | *Indica* | SG3 |
| 21 | Cocodrie | Louisiana | *Japonica* | SG7 | 56 | SLO16 | India | *Japonica* | SG3 |
| 22 | R609 | Louisiana | *Indica* | SG3 | 57 | Djogolon | Burkina Faso | *Indica* | SG3 |
| 23 | LAH10 | Louisiana | *Japonica* | SG3 | 58 | Colusa | Louisiana | *Japonica* | AD |
| 24 | LA0802140 | Louisiana | *Japonica* | SG7 | 59 | Acadia | Louisiana | *Japonica* | NG |
| 25 | Cheniere | Louisiana | *Japonica* | SG7 | 60 | Delitus-1206 | Louisiana | *Japonica* | SG5 |
| 26 | Bengal | Louisiana | *Japonica* | SG4 | 61 | Tokalon | Louisiana | *Japonica* | AD |
| 27 | CL152 | Louisiana | *Japonica* | SG7 | 62 | Evangeline | Louisiana | *Japonica* | SG5 |
| 28 | Roy J | Louisiana | *Japonica* | AD | 63 | Pirogue | Louisiana | *Japonica* | SG4 |
| 29 | Rey | Louisiana | *Japonica* | SG6 | 64 | Rexona | Louisiana | *Japonica* | SG3 |
| 30 | CL142 | Louisiana | *Japonica* | SG6 | 65 | Nira | Louisiana | *Japonica* | SG5 |
| 31 | Mermentau | Louisiana | *Japonica* | SG7 | 66 | Magnolia | Louisiana | *Japonica* | AD |
| 32 | Jupiter | Louisiana | *Japonica* | SG4 | 67 | Lacrosse | Louisiana | *Japonica* | SG4 |
| 33 | Wells | Arkansas | *Japonica* | SG6 | 68 | Sunbonnet | Louisiana | *Japonica* | SG6 |
| 34 | Catahoula | Louisiana | *Japonica* | SG7 | 69 | Ecrevisse | Louisiana | *Japonica* | AD |
| 35 | Kalia | Bangladesh | *Indica* | SG7 | 70 | Toro | Louisiana | *Japonica* | SG6 |

| S.N. | Genotype | Source | Sub-type | Sub-group | S.N. | Genotype | Source | Sub-type | Sub-group |
| --- | --- | --- | --- | --- | --- | --- | --- | --- | --- |
| 71 | Nato | Louisiana | *Japonica* | AD | 106 | Rexark Rogue_9262 | Texas | *Japonica* | SG1 |
| 72 | Saturn | Louisiana | *Japonica* | AD | 107 | Smooth Zenith | Texas |  | SG1 |
| 73 | Della | Louisiana | *Japonica* | SG6 | 108 | Short Century | Texas | *Japonica* | SG2 |
| 74 | Vista | Louisiana | *Japonica* | SG4 | 109 | Family 24 | Arkansas | *Japonica* | SG1 |
| 75 | Trenasse | Louisiana | *Japonica* | AD | 110 | Century Patna | Texas | *Japonica* | SG2 |
| 76 | LA110 | Louisiana | *Japonica* | SG3 | 111 | Early Colusa | California | *Japonica* | SG8 |
| 77 | Leah | Louisiana | *Japonica* | SG5 | 112 | Rexark Rogue_9214 | Texas | *Japonica* | AD |
| 78 | Toro-2 | Louisiana | *Japonica* | NG | 113 | Century Rogue | Texas | *Japonica* | SG2 |
| 79 | Mercury | Louisiana | *Japonica* | SG4 | 114 | Nira 43 | Texas | *Japonica* | AD |
| 80 | Lacassine | Louisiana | *Japonica* | AD | 115 | Arkose Selection | Arkansas | *Japonica* | AD |
| 81 | Jodon | Louisiana | *Japonica* | AD | 116 | Pecos | Texas | *Japonica* | SG1 |
| 82 | Dellrose | Louisiana | *Japonica* | SG6 | 117 | Skybonnet | Texas | *Japonica* | SG6 |
| 83 | Lafitte | Louisiana | *Japonica* | SG4 | 118 | Tebonnet | Arkansas | *Japonica* | SG6 |
| 84 | Dellmati | Louisiana | *Japonica* | SG7 | 119 | M-202 | California | *Japonica* | SG8 |
| 85 | Earl | Louisiana | *Japonica* | SG4 | 120 | M-102 | California | *Japonica* | SG8 |
| 86 | Della-2 | Louisiana | *Japonica* | SG7 | 121 | Rico 1 | Texas | *Japonica* | AD |
| 87 | Gulfrose | Texas | *Japonica* | AD | 122 | M-103 | California | *Japonica* | SG8 |
| 88 | R27 | Missouri | *Japonica* | SG4 | 123 | Katy | Arkansas | *Japonica* | SG2 |
| 89 | Starbonnet | Arkansas | *Japonica* | AD | 124 | S-301 | California | *Japonica* | SG8 |
| 90 | Zenith | Arkansas | *Japonica* | AD | 125 | Maybelle | Texas | *Japonica* | SG1 |
| 91 | Rexark | Arkansas | *Japonica* | SG3 | 126 | Sierra | Texas | *Japonica* | SG6 |
| 92 | Earlirose | California | *Japonica* | SG8 | 127 | Lotus | Texas | *Japonica* | SG6 |
| 93 | Caloro | California | *Indica* | AD | 128 | Neches | Texas | *Japonica* | SG6 |
| 94 | Gody | California | *Japonica* | SG4 | 129 | Carolina Gold | Texas | *japonica* | SG1 |
| 95 | Bond | Arkansas | *Japonica* | AD | 130 | Presidio | Texas | *Japonica* | SG1 |
| 96 | Newbonnet | Arkansas | *Japonica* | SG6 | 131 | Sabine | Texas | *Japonica* | AD |
| 97 | Vegold | Arkansas | *Japonica* | SG6 | 132 | Lavaca | Texas | *Japonica* | SG6 |
| 98 | Gold Zenith | Arkansas | *Japonica* | AD | 133 | MS-1995-15 | Mississippi | *Japonica* | SG3 |
| 99 | Belle Patna | Texas | *Japonica* | SG6 | 134 | MS-1996-9 | Mississippi | *Japonica* | SG3 |
| 100 | Nova | Arkansas | *Japonica* | AD | 135 | Delitus | Louisiana | *Japonica* | AD |
| 101 | Palmyra | Missouri | *Japonica* | SG1 | 136 | Salvo | Louisiana | *Japonica* | AD |
| 102 | Nova 66 | Arkansas | *Japonica* | AD | 137 | Stormproof | Arkansas | *Japonica* | SG1 |
| 103 | Glutinous Zenith | Texas | *Japonica* | SG3 | 138 | Calady | California | *Japonica* | SG8 |
| 104 | Dawn | Texas | *Japonica* | SG2 | 139 | Zenith-2 | Arkansas | *Japonica* | SG1 |
| 105 | Bluebelle | Texas | *Japonica* | SG6 | 140 | Arkansas Fortuna | Arkansas | *japonica* | SG2 |

| S.N. | Genotype | Source | Sub-type | Sub-group | S.N. | Genotype | Source | Sub-type | Sub-group |
| --- | --- | --- | --- | --- | --- | --- | --- | --- | --- |
| 141 | Arkrose | Arkansas | *japonica* | SG8 | 174 | Terso | California | Japonica | SG8 |
| 142 | Prelude | Arkansas | *Japonica* | SG2 | 175 | Texas Patna | Texas | Japonica | SG1 |
| 143 | Asahi | Arkansas | *Japonica* | SG8 | 176 | Bluebonnet | Texas | Japonica | SG2 |
| 144 | Kamrose | Arkansas | *Japonica* | SG8 | 177 | Cody | California | Japonica | SG2 |
| 145 | Newrex | Texas | *Japonica* | AD | 178 | RD | Texas | Japonica | SG6 |
| 146 | M-301 | California | *Japonica* | SG8 | 179 | Rexark-2 | Arkansas | Japonica | SG1 |
| 147 | S-201 | California | *japonica* | SG8 | 180 | Calrose-2 | California | japonica | SG8 |
| 148 | M-401 | California | *Japonica* | SG8 | 181 | TP 49 | Texas | Japonica | AD |
| 149 | M-302 | California | *Japonica* | SG8 | 182 | SP 14 | Texas | Japonica | AD |
| 150 | Bellemont | Texas | *Japonica* | AD | 183 | C-4 | Texas | Japonica | SG1 |
| 151 | M-201 | California | Japonica | AD | 184 | Hill Long Grain | Texas | Japonica | AD |
| 152 | Northrose | Arkansas | Indica | AD | 185 | Nortai | Arkansas | Japonica | AD |
| 153 | Calrose | California | japonica | SG2 | 186 | Brazos | Texas | Japonica | AD |
| 154 | Bluebelle-2 | Texas | Japonica | SG2 | 187 | Lebonnet | Texas | Japonica | SG2 |
| 155 | Lady Wright | Arkansas | Japonica | AD | 188 | Saturn Rogue | Arkansas | Japonica | SG2 |
| 156 | Early Prolific | Arkansas | Japonica | SG1 | 189 | Mars | Arkansas | Japonica | AD |
| 157 | Hybrid Mix | Texas | Japonica | AD | 190 | Starbonnet | Arkansas | Japonica | AD |
| 158 | Hill medium | Texas | Japonica | SG3 | 191 | Gold Nato | Arkansas |  | SG2 |
| 159 | Glutinous Selection | Texas | Japonica | SG2 | 192 | Earlirose-2 | California | Japonica | NG |
| 160 | R-50 | Missouri | Japonica | SG2 | 193 | Early Wataribur | California | Japonica | SG8 |
| 161 | MO R-500 | Missouri | Japonica | AD | 194 | Conway | California | Japonica | NG |
| 162 | R-54 | Missouri | Japonica | SG2 | 195 | Texmont | Texas | Japonica | NG |
| 163 | R-52 | Missouri | Japonica | AD | 196 | Alan | Arkansas | Japonica | NG |
| 164 | R-27-1 | Missouri | Japonica | NG | 197 | Millie | Arkansas | Japonica | NG |
| 165 | Jefferson | Texas | Japonica | SG2 | 198 | Dellmont | Texas | Japonica | NG |
| 166 | Melrose | Texas | Japonica | AD | 199 | Rosemont | Texas | Japonica | NG |
| 167 | Dixiebelle | Texas | Japonica | AD | 200 | Orion | Arkansas | Japonica | NG |
| 168 | Jasmine 85 | Texas | Indica | AD | 201 | M-204 | California | Japonica | SG8 |
| 169 | Carlpearl | California | Japonica | AD | 202 | Adair | Arkansas | Japonica | NG |
| 170 | Madison | Texas | Japonica | SG6 | 203 | LaGrue | Arkansas | Japonica | NG |
| 171 | Tsuri Mai | California | Japonica | SG8 | 204 | Jackson | Texas | Japonica | NG |
| 172 | Kokubelle | California | Japonica | AD | 205 | Azucena | Philippines | Indica | NG |
| 173 | Maxwell | California | Japonica | SG8 |  |  |  |  |  |

^a^source where the variety was developed, ^b^*Indica* or *japonica* subtype, ^c^ Sub-group classification by ‘Structure’ software (SG: Subgroup, AD: Admixture, NG: Not genotyped)
