# Supplementary figures and images for: Genetic Diversity, Population Structure, and Marker-Trait Association for Drought Tolerance in US Rice Germplasm
Source: Plants (Basel). 2019 Nov 21;8(12):530. doi: 10.3390/plants8120530 (PMC6963191; doi:10.3390/plants8120530)

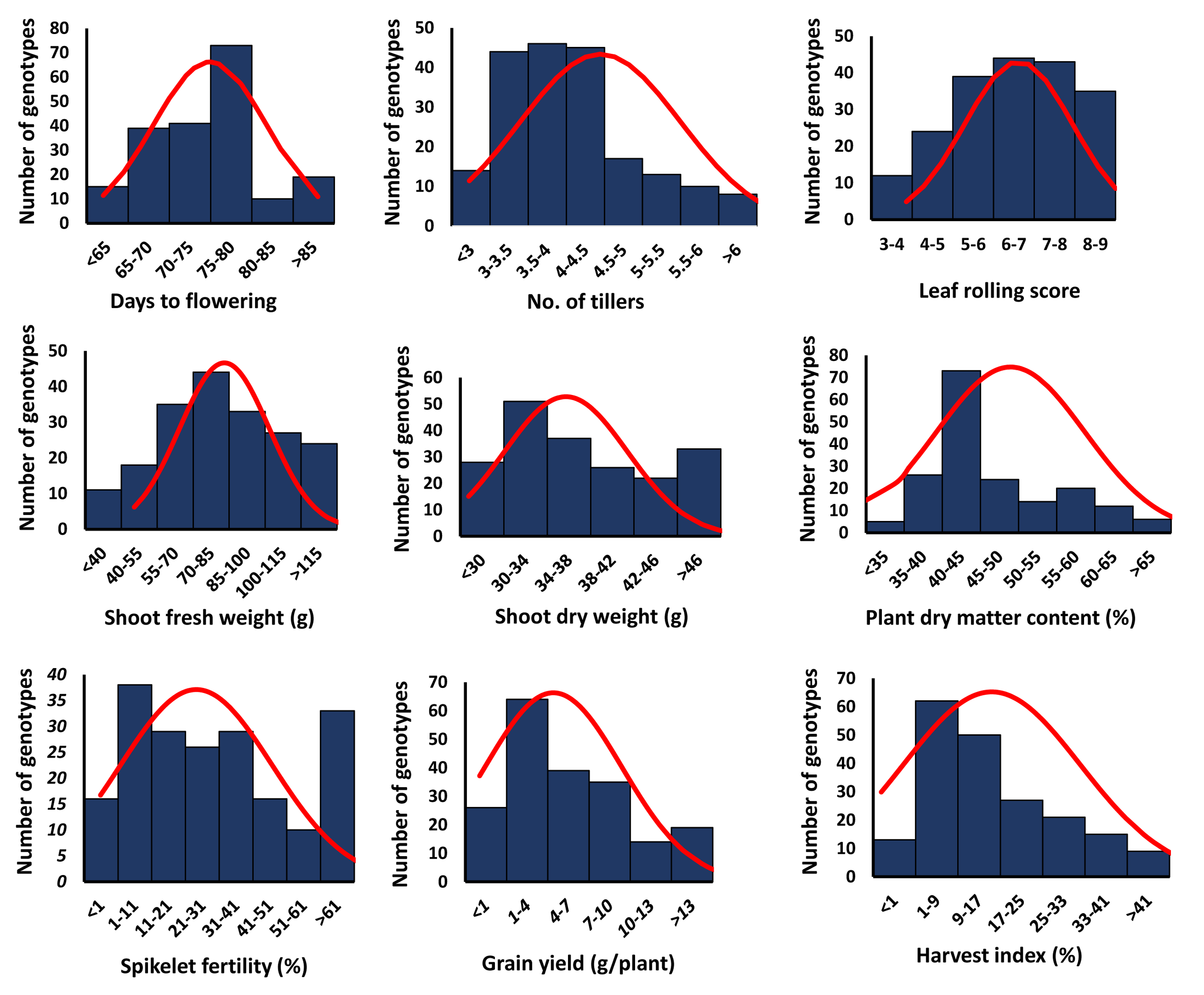

Supplement: Supplementary file 1 [file plants-08-00530-s001.zip › Supplementary Figure S1.tif]

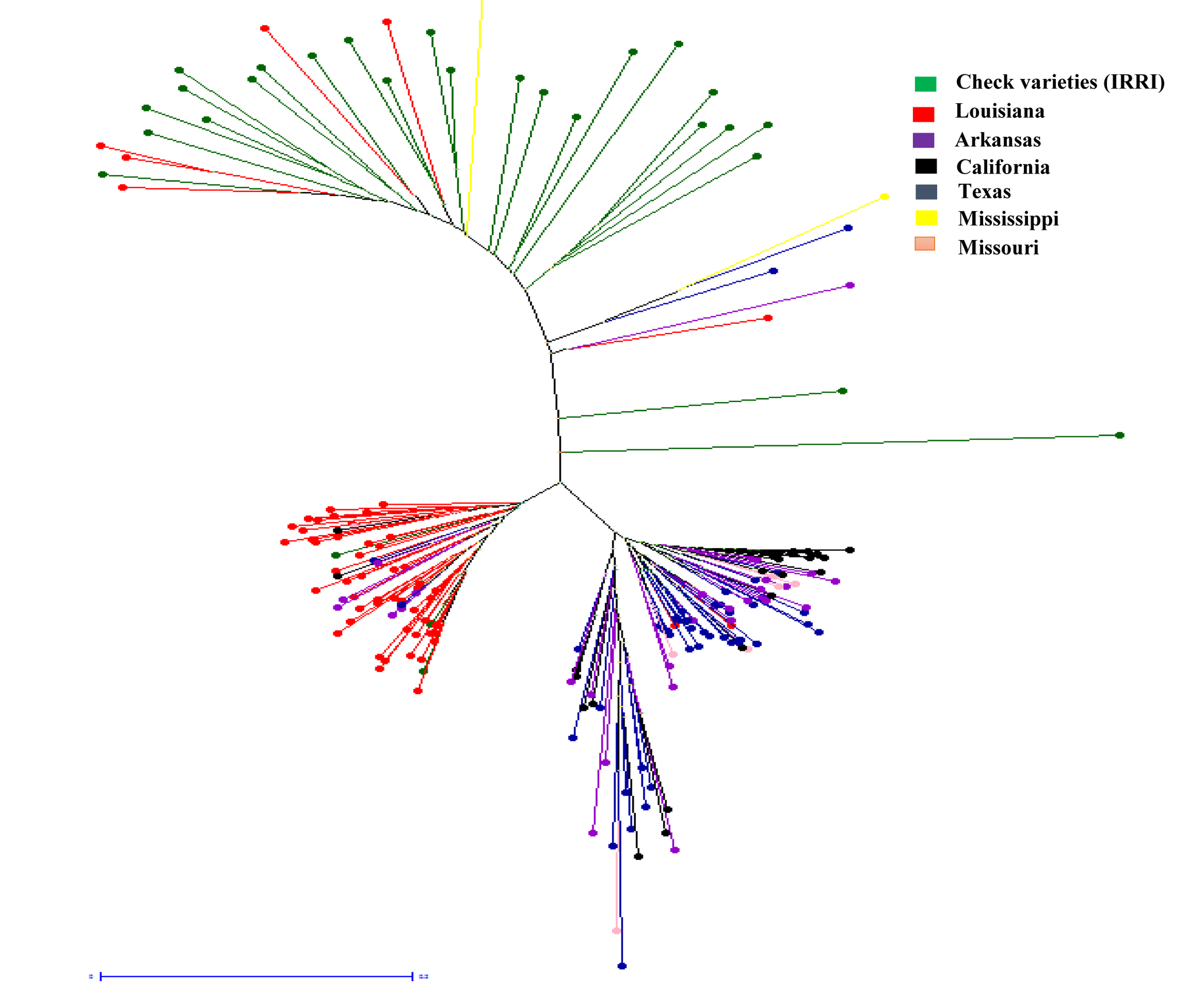

Supplement: Supplementary file 1 [file plants-08-00530-s001.zip › Supplementary Figure S3.tif]

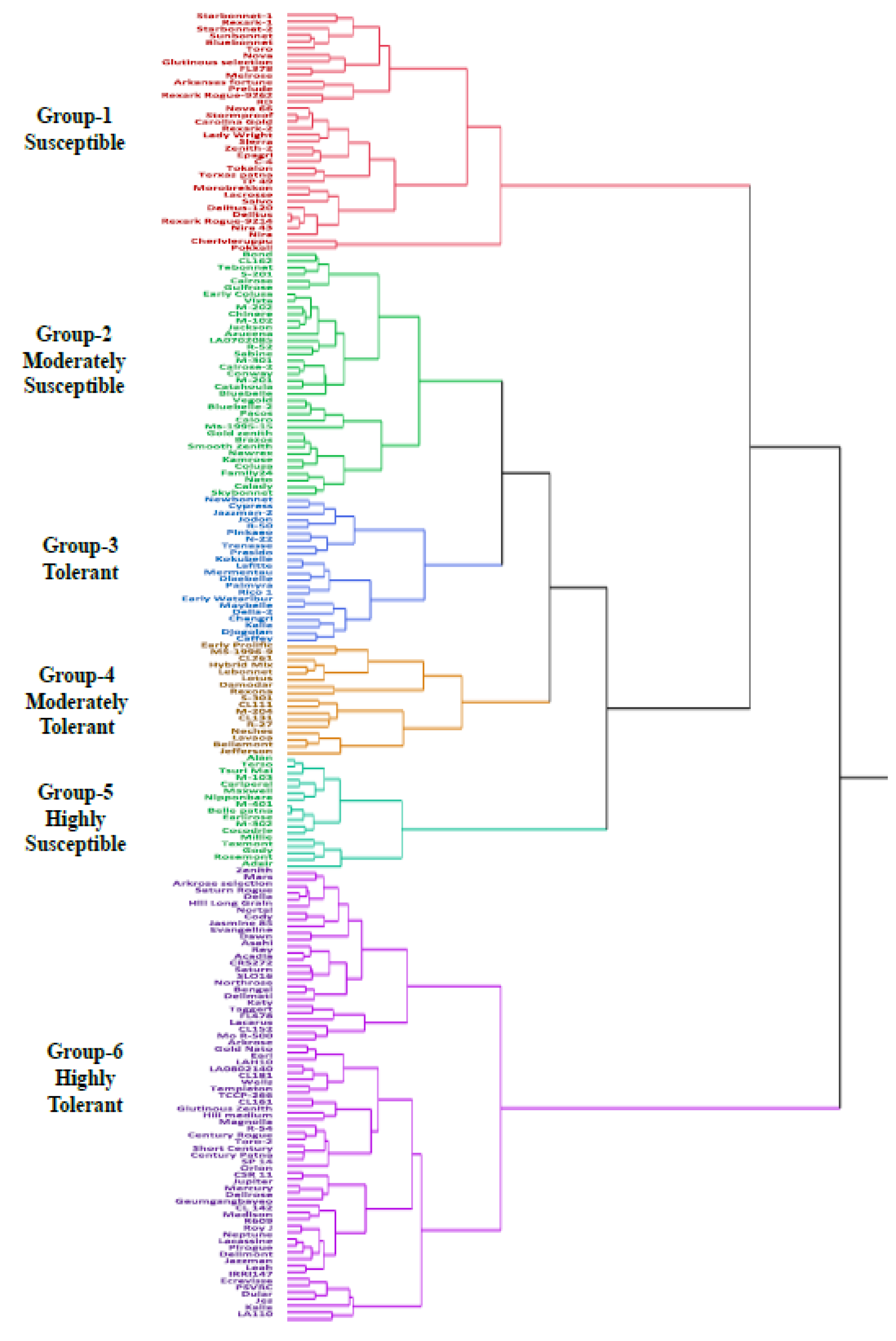

Supplement: Supplementary file 1 [file plants-08-00530-s001.zip › Supplementary Figure-S2.tif]
